# Enhancing the Performance of Si/Ga_2_O_3_ Heterojunction Solar-Blind Photodetectors for Underwater Applications

**DOI:** 10.3390/nano15141137

**Published:** 2025-07-21

**Authors:** Nuoya Li, Zhixuan Liao, Linying Peng, Difei Xue, Kai Peng, Peiwen Lv

**Affiliations:** 1 College of Chemistry, Fuzhou University, Fuzhou 350108, China; linuoya@fjirsm.ac.cn (N.L.); liaozhixuan@fjirsm.ac.cn (Z.L.); penglinying@fjirsm.ac.cn (L.P.); 2 State Key Laboratory of Functional Crystals and Devices Fujian Institute of Research on the Structure of Matter, Chinese Academy of Sciences, Fuzhou 350002, China; xuedifei@fjirsm.ac.cn; 3 Fujian College, University of Chinese Academy of Sciences, Fuzhou 350002, China; pengkai@fjirsm.ac.cn

**Keywords:** solar-blind ultraviolet, photodetector, self-power, Ga_2_O_3_ nanostructures

## Abstract

Epitaxial growth of β-Ga_2_O_3_ nanowires on silicon substrates was realized by the low-pressure chemical vapor deposition (LPCVD) method. The as-grown Si/Ga_2_O_3_ heterojunctions were employed in the Underwater DUV detection. It is found that the carrier type as well as the carrier concentration of the silicon substrate significantly affect the performance of the Si/Ga_2_O_3_ heterojunction. The p-Si/β-Ga_2_O_3_ (2.68 × 10^15^ cm^−3^) devices exhibit a responsivity of up to 205.1 mA/W, which is twice the performance of the devices on the n-type substrate (responsivity of 93.69 mA/W). Moreover, the devices’ performance is enhanced with the increase in the carrier concentration of the p-type silicon substrates; the corresponding device on the high carrier concentration substrate (6.48 × 10^17^ cm^−3^) achieves a superior responsivity of 845.3 mA/W. The performance enhancement is mainly attributed to the built-in electric field at the p-Si/n-Ga_2_O_3_ heterojunction and the reduction in the Schottky barrier under high carrier concentration. These findings would provide a strategy for optimizing carrier transport and interface engineering in solar-blind UV photodetectors, advancing the practical use of high-performance solar-blind photodetectors for underwater application.

## 1. Introduction

Solar-blind photodetectors (SBPDs) exploit their insensitivity to sunlight within the 200–280 nm spectral band, enabling critical applications in space security communications, ozone layer monitoring, missile early warning, and underwater target identification [1,2,3,4]. SBPDs are uniquely indispensable for underwater detection due to their inherent immunity to solar background noise, selective sensitivity to trace DUV signatures, and compatibility with the deep ultraviolet transmission window in aquatic environments. As a result, SBPDs are increasingly recognized as a key component in the development of next-generation marine exploration systems, autonomous underwater vehicles (AUVs), and underwater photodetectors [5,6,7,8].

In addition to spectral selectivity, the materials used in the underwater detection must also exhibit strong chemical stability and corrosion resistance in aqueous solutions and typically ion-rich environments. Due to their resistance to hydrolysis and surface oxidation, materials with strong chemical inertia (such as diamond, AlGaN alloys, and β-Ga_2_O_3_) are preferred [9]. Distinct from solid-state counterparts, underwater photodetectors necessitate fully autonomous operation without external electrodes or power inputs, mandating robust self-powered characteristics in device design [10,11,12].

Gallium oxide (Ga_2_O_3_) is a novel ultra-wideband semiconductor material characterized by high absorption coefficients and excellent stability. Its direct bandgap is approximately 4.9 eV, located in the solar-blind region. Compared with other candidate materials such as diamond and AlGaN, β-Ga_2_O_3_ combines an exceptional chemical inertness with a uniquely wide bandgap (4.8 eV), yielding intrinsically solar-blind deep-UV selectivity without the need for complex heterostructuring or expensive substrates. Additionally, β-Ga_2_O_3_ exhibits high transparency in the visible light spectrum and low growth costs, making it one of the most suitable materials for manufacturing SBUV detectors [13,14]. Chen et al. recently reported a photodiode (PD) based on an α-Ga_2_O_3_ nanorod array (NRA), with a response/recovery time (τ_r_/τ_d_ = 0.43/0.17 s) and responsivity of 1.44 mA/W [15]. Huang et al. further developed α-Ga_2_O_3_ NRAs PDs, which increased the responsivity to 11.34 mA/W, with τ_r_/τ_d_ values of 1.51/0.18 s [16]. Feng et al. synthesized an array of β-Ga_2_O_3_ nanorods coated with an amorphous Ga_2_O_3_ layer on a silicon substrate, with a response rate of 48.4 mA/W and a response/recovery time of 0.12/0.16 s [17]. Existing research has made significant progress in material and device optimization [18,19]. However, compared to traditional SBPDs, the performance of underwater devices remains relatively limited, largely due to the lack of systematic investigation into the impact of substrate material properties on device performance.

Here, we present the growth of β-Ga_2_O_3_ nanowires (NARs) with good crystallinity on silicon substrates with different doping types and doping concentrations by a simple CVD technique. Subsequently, DUV PDs based on β-Ga_2_O_3_ NRAs were prepared, and the obtained PDs exhibited self-powered solar-blind DUV detection with a high responsivity of 845.3 mA/W at 254 nm and 0.6 mW/cm^2^ light intensity. Then, the reliability of the prepared β-Ga_2_O_3_ PDs is explored. These results indicate that β-Ga_2_O_3_ nanostructures are promising candidates for low-cost, self-powered, high-sensitivity solar-blind DUV PD applications in next-generation sustainable integrated optoelectronic systems.

## 2. Materials and Methods

**Figure 1:**
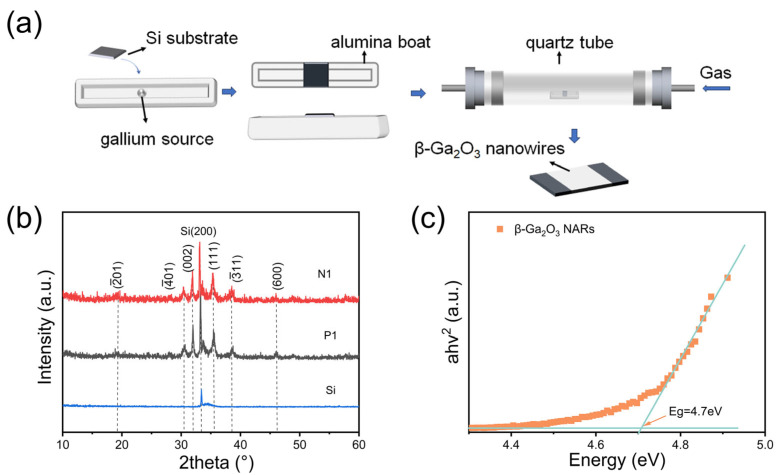
(**a**) Growth schematic of β-Ga_2_O_3_ NARs grown on silicon substrates at 900 °C for 30 min. (**b**) XRD spectrum. (**c**) (ahv)^2^-hv plot.

β-Ga_2_O_3_ NARs were epitaxially grown on silicon substrates by the LPCVD technique. High-purity gallium (UMC, Hsinchu, Taiwan, 99.99999%) and oxygen (O_2_, 99.9%) were used as raw material precursors, and argon (Ar, 99.999%) was used as the carrier gas for the experiments. Figure 1a demonstrates a typical preparation process for β-Ga_2_O_3_ PDs. The specific process parameters were as follows: the gallium source mass was 10.0 mg, the reaction chamber pressure was set at 15 Torr, and the oxygen flow rate was precisely controlled at 20.0 sccm.

Substrate pre-treatment stage: One-sided polished (100) crystal-oriented silicon substrate (size 10 mm × 10 mm) was selected and immersed in 5% HF dilution for 5 min to remove the surface oxide layer. After immersion, the silicon substrate was rinsed with deionized water and ultrasonicated with acetone, deionized water, and ethanol for 10 min. The gold film was deposited via magnetron sputtering at room temperature for 10 s, yielding a uniform thickness of 5.0 ± 0.5 nm. The treated substrate was placed in a ceramic boat (size 8 cm × 1.2 cm) directly above the gallium source.

Growth process control: The tube furnace chamber was first pumped to a high vacuum (<10^−2^ Torr), followed by the introduction of high-purity argon at a constant flow rate (100 sccm) as a protective atmosphere. The temperature was rapidly increased at a rate of 20 °C/min to 710 °C, then slowly increased to 752 °C, and a 30 min gas phase reaction was carried out while introducing Ar (100 sccm) and O_2_ (20.0 sccm). At the end of growth, the atmosphere was switched to pure argon atmosphere (100 sccm) and naturally cooled to room temperature.

Substrate parameter design: n-type and p-type doped silicon substrates are selected for the experiment. Specifically, they are divided into four groups (P1 (p-type, 2.68 × 10^15^ cm^−3^), P2 (p-type, 3.19 × 10^16^ cm^−3^), P3 (p-type, 6.48 × 10^17^ cm^−3^), and N1 (n-type, 8.95 × 10^14^ cm^−3^)) to systematically study the effects of the substrate carrier type and carrier concentration on the performance of the heterojunction.

The synthesized samples were characterized by X-ray diffraction (XRD) to determine crystal structures and phase purity, and scanning electron microscopy (SEM) to analyze surface morphology. The XRD patterns were collected using a high-resolution X-ray diffractometer at Rigaku Smart Laboratory, Tokyo, Japan. The samples were scanned in the range of 10–60° using a scanning speed of 2°/min. The surface elemental compositions of Ga_2_O_3_ nanowires were characterized and performed with an X-ray photoelectron spectrometer (XPS) model ESCALAB Xi+ from Thermo Fisher, Waltham, MA, USA. Al Kα rays (energy 1486.68 eV) were used as the excitation source and the X-ray spot diameter was 650 μm. The elements scanned were C, Si, Ga, and O. Binding energies were scanned in the range 0–1400 eV in 0.1 eV steps. The data were then analyzed using the Avantage program and all XPS binding energies were calibrated to the C 1s peak at 284.8 eV before peak fitting, and the appropriate charge correction was applied to obtain accurate binding energy values. SEM images were taken at 15.0 kV using a HITACHIUHR SU8010, Tokyo, Japan. The UV transmission spectra of β-Ga_2_O_3_ NARs were studied using a UV–visible spectrophotometer (UV-2550) in the wavelength range of 200–600 nm. It was tested in a three-electrode quartz cell with high DUV transmittance by an electrochemical workstation (CHI440C, Shanghai Chenhua Co., Ltd., Shanghai, China). A silicon substrate on which β-Ga_2_O_3_ NARs were grown was used as the working electrode. A platinum sheet (1 × 1 cm^2^) and an Ag/AgCl electrode (RHE) were chosen as the counter electrode and reference electrode, respectively. The test environment was 0.5 M Na_2_SO_4_ aqueous solution (weakly alkaline), and the UVC light source was provided by low-pressure lamps with wavelengths of 254 nm and 365 nm.

## 3. Result and Discussion

Figure 1b shows the XRD patterns of β-Ga_2_O_3_ NARs grown on the n-type silicon substrate (N1) and p-type silicon substrate (P1), respectively, in which there are multiple diffraction peaks of β-Ga_2_O_3_, among which, the diffraction peaks located at 18.9°, 30.5°, 31.7°, 35.2°, 38.4°, and 45.8° correspond to the β-Ga_2_O_3_ (201), (401), (002), (111), (311), and (600) diffraction surfaces, and these peaks confirm the formation of β-Ga_2_O_3_ (PDF#76-0573). The peak at 33.4° is confirmed to be the diffraction peak of the Si substrate (200) (PDF#27-1402). As can be seen from the figure, there is no significant difference in the peak positions and peak intensities between the two samples, and the difference in the half peak widths (FWHM) is less than 5%, which confirms that the different doping types of the substrate have a negligible effect on the crystal growth kinetics of β-Ga_2_O_3_. The UV–Vis transmission spectrum of β-Ga_2_O_3_ NARs is shown in Figure 1c, which was fitted to the direct bandgap semiconductor based on Tauc’s formula: ${\left(\alpha h\vartheta \right)}^{2}=A(h\vartheta -E_{g})$, where α is the absorption coefficient, hν is the photon energy, and A is the proportionality constant. By linearly extrapolating the intercept of the ${\left(\alpha h\vartheta \right)}^{2}$ versus the hϑ curve, the optical bandgap of β-Ga_2_O_3_ NARs is determined to be 4.70 ± 0.05 eV.

**Figure 2:**
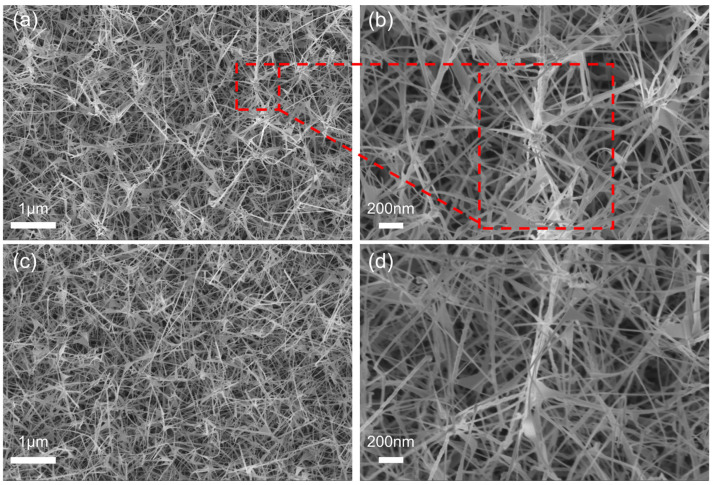
β-Ga_2_O_3_ nanowires: P1 sample (**a**) low magnification view and (**b**) high magnification view. N1 sample (**c**) low magnification view and (**d**) high magnification view.

The surface morphology of β-Ga_2_O_3_ NARs was verified by SEM (shown in Figure 2), and it can be seen that the samples are in the state of nanowires and have a homogeneous diameter of about 20 nm, with lengths ranging from a few micrometers to a dozen micrometers, The nanowires are cross-connected in a random orientation and form a cross-linked structure at the contact points. The red box is the cross-linked part. Cross-linking facilitates carrier transport by establishing an extensive conductive network, thereby improving electrical performance. It is noteworthy that the substrate carrier type has a negligible effect on the kinetic process of the gas–liquid–solid (VLS) growth mechanism. This stable morphology provides a material basis for large-scale integrated optoelectronic devices.

**Figure 3:**
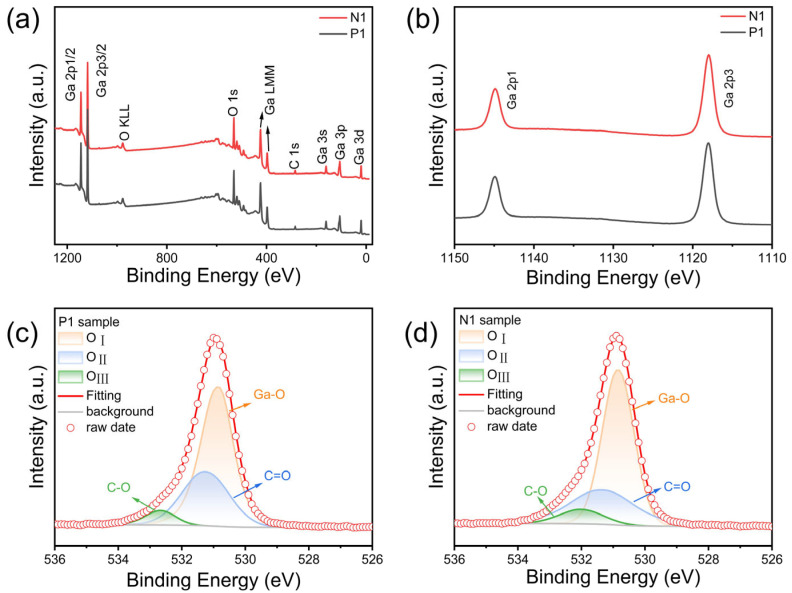
XPS spectra of β-Ga_2_O_3_ NARs: (**a**) measured spectra of P1 and N1 samples. (**b**) Ga 2p spectra. O 1s spectra of (**c**) P1 sample and (**d**) N1 sample.

To understand the surface chemical state and composition of β-Ga_2_O_3_, we performed an XPS study on samples N1 and P1, as shown in Figure 3. The XPS was calibrated by measuring the power functions of the samples. The C 1s peak as a reliable binding energy (BE) reference depends on the power function ($\emptyset_{SA}$) of the samples [20]. The value of $\emptyset_{SA}$ is related to the following equation: $\emptyset_{SA}=h\vartheta +E_{cutoff}-E_{F}$, where $E_{cutoff}$ is the fitted value of the cut-off edge, $E_{F}$ is the Fermi energy level, and the β-Ga_2_O_3_ power function is calculated to be 4.1 eV [21]. In addition, the position of the C1s peaks was calibrated to be 285.48 eV [22,23] according to the equation $E_{B}^{F}=289.58-\emptyset_{SA}$. It is clear from Figure 3a that the elemental peaks other than the C, Ga, and O peaks were not detected in the XPS measurement spectra, indicating the high purity of the grown β-Ga_2_O_3_ nanowires. Figure 3b shows the Ga 2p spectra of the two samples. It is observed that the binding energy peaks of Ga 2p1/2 and Ga 2p3/2 are separated by 27.0 eV, which is consistent with the binding energy of Ga 2p [24,25]. Figure 3c,d shows the division of the O1s core energy level spectra into three components by Gaussian fitting analysis. These components are lattice oxygen (O_I_), i.e., the oxygen atom bonded to the gallium atom in β-Ga_2_O_3_, which represents the oxygen environment of the material proper; O^2−^ (O_II_) in the oxygen-deficient region; and carbonate and hydroxyl-(O_III_), which corresponds to the chemical adsorption on the surface of the film, respectively [26,27]. The density of oxygen vacancies is determined by the intensity ratio of O_II_/(O_I_ + O_II_). The calculated intensity ratios of P1 and N1 are 31.03% and 30.07%, respectively.

**Figure 4:**
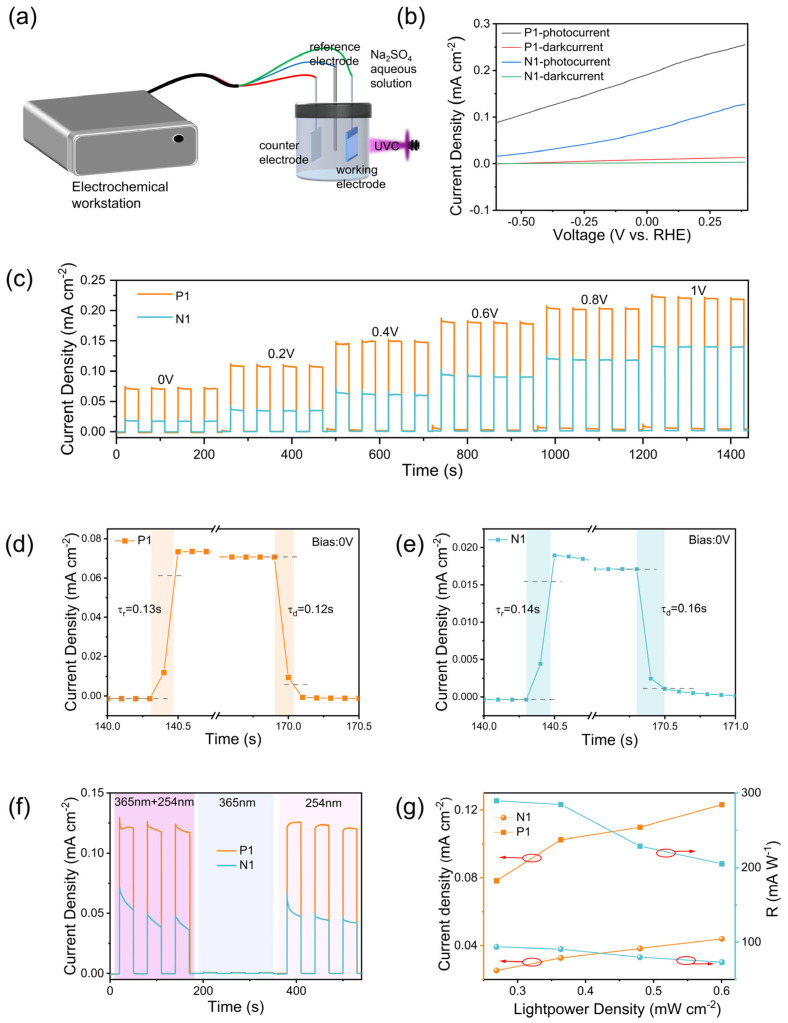
(**a**) Schematic diagram of the simulated underwater detector evaluation system for β-Ga_2_O_3_ nanowires. (**b**) Linear voltage–current (LSV) curves of N1 and P1 samples under 0–1 V bias. (**c**) I-V response curves under different biases. Rise and decay times of P1 (**d**) and N1 (**e**) samples. (**f**) Photocurrent density under different wavelengths of light. (**g**) Fitted curves of photoluminescent devices under different light intensities and corresponding photoresponse calculations.

The similar morphology and XPS spectra observed in both samples indicate good experimental repeatability and reliability. Furthermore, the carrier type of the silicon substrate exerts negligible influence on the film structure and electrochemical properties of the β-Ga_2_O_3_ NARs. Similar oxygen vacancy concentrations may result in similar sample performance, given the established correlation between oxygen vacancies and device characteristics [28,29]. However, electrochemical measurements reveal significant performance discrepancies among p-n junction devices. Figure 4a shows a schematic of the experimental test configuration based on the three-electrode system, and all electrochemical characterizations were conducted at constant temperature (25 ± 0.5 °C). Notably, at 254 nm illumination and 0 V bias (Figure 4b), sample P1 exhibits a current density approximately fivefold higher than sample N1. When the bias voltage increases from 0 V to 1 V (−0.6~0.4 V vs. RHE), the photocurrent density of the P1 detector rises sharply to 0.25 mA/cm^2^—two orders of magnitude above its dark current. This demonstrates that the fabricated PDs operate effectively in self-powered mode (0 V) while maintaining excellent optoelectronic responsivity. Given the pronounced sensitivity of semiconductor/electrolyte photodetectors to external parameters, the impact of applied bias voltage on device performance was systematically investigated. As depicted in Figure 4c, both P1 and N1 detectors exhibit defined on/off switching characteristics across a 0–1 V bias range at 30 s intervals, demonstrating high signal reproducibility. A quantitative relationship between photocurrent enhancement and increasing bias voltage is observed, indicating improved separation efficiency of photogenerated carriers at elevated electric fields. Consequently, systematic modulation of the bias voltage serves as a critical factor for optimizing detection performance [30].

The rise time (τ_r_) and decay time (τ_d_) are defined as the time required for the photocurrent to rise to 90% and fall to 10% of the peak value, respectively [31]. As evidenced in Figure 4d,e, both samples demonstrate rapid response kinetics (τ_r_, τ_d_< 0.2 s at 0 V bias), with the P1 device exhibiting marginally faster response times (τ_r_, τ_d_ < 0.15 s) compared to N1. Figure 4f presents the characteristic self-powered switching behavior under varied illumination wavelengths (0 V bias). Notably, both devices exhibit no detectable photoresponse under 365 nm irradiation. Conversely, abrupt photoresponse onset occurs upon 254 nm UV illumination, where the photocurrent rapidly saturates at 0.12 mA/cm^2^ (P1 sample). Crucially, concurrent 365/254 nm illumination yields identical response characteristics to 254 nm irradiation alone. To quantify this selectivity, we introduce the rejection ratio: $R_{rej}=\frac{I_{254\;nm}}{I_{365\;nm}}$, where I_254 nm_ and I_365 nm_ are the steady-state photocurrents under 254 nm and 365 nm illumination, respectively, at the same optical power density. The results are shown in Table 1. These results collectively validate pronounced solar-blind UV selectivity in the fabricated devices [16,17]. As a fundamental performance metric for photodetectors, the photocurrent is defined as the incremental current generated by photogenerated carriers. Figure 4g presents the characteristic self-powered switching behavior under varied light intensities (0 V bias) and corresponding photoresponse calculations. This linear dependence further indicates the efficient separation of photoexcited electron–hole pairs with minimal trap-state involvement [32,33]. The photoresponsivity (R) is defined as the ratio of the photocurrent I_ph_ to the incident light power P_in_. This parameter reflects the efficiency of the photodetector in converting incident light into an electrical signal. Both devices exhibit peak responsivity values at low light intensity (0.27 mW/cm^2^): 289.7 mA/W (P1) and 93.69 mA/W (N1). At 0.6 mW/cm^2^, responsivities decrease to 205.1 mA/W (P1) and 73.13 mA/W (N1), respectively. This responsivity reduction at higher intensities is attributed to increased photogenerated carrier density, which elevates bimolecular recombination rates and consequently reduces photon utilization efficiency [34]. The three times responsivity disparity between P1 and N1 samples at 0.6 mW/cm^2^ illumination intensity is attributed to the fundamental operational principle of pn-junction self-powered photodetectors, where the depletion layer serves as the primary carrier transport region. Enhanced separation efficiency and accelerated extraction kinetics of photogenerated carriers occur under larger built-in potentials. Consequently, Si/Ga_2_O_3_ pn-junction devices demonstrate superior self-powered performance compared to nn-junction counterparts [35].

**Figure 5:**
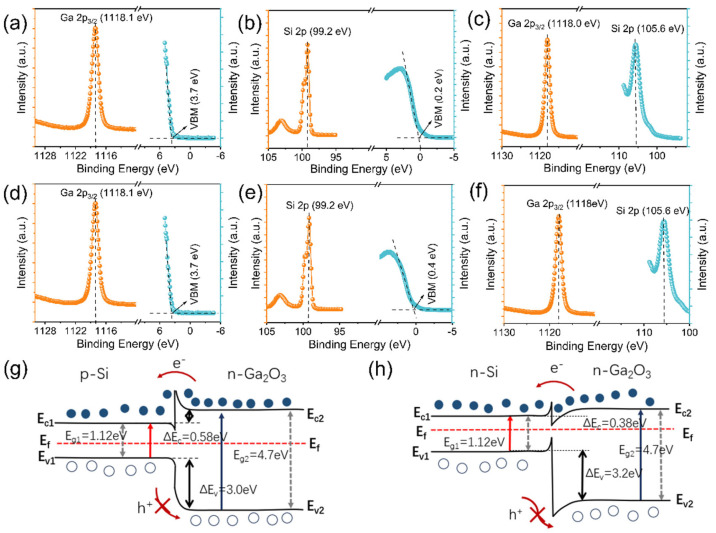
Ga 2p3/2, VBM (**a**) and Si 2p, VBM (**b**) and Ga 2p3/2 and Si 2p core level spectrum (**c**) of P1 sample. Ga 2p3/2, VBM (**d**) and Si 2p, VBM (**e**) and Ga 2p3/2 and Si 2p core level spectrum (**f**) of N1 sample. Energy band schematic of the (**g**) P1 sample and (**h**) N1 sample.

To validate the theoretical framework, XPS and UPS analyses were conducted on both samples (Figure 5). The valence band offset ($∆E_{v}$) at the Ga_2_O_3_/Si heterojunction was determined via Kraut’s method [36,37]:${\;∆E}_{v}={∆E}_{CL}+\left(E_{Si2p}^{Si}-E_{VBM}^{Si}\right)-(E_{Ga2p_{3/2}}^{{Ga}_{2}O_{3}}-E_{VBM}^{Ga_{2}O_{3}})$, where $∆E_{CL}=(E_{Ga2p_{3/2}}^{{Ga}_{2}O_{3}}-E_{Si2p}^{Si})$ denotes the core-level (CL) energy difference measured at the heterointerface. The terms $\left(E_{Si2p}^{Si}-E_{VBM}^{Si}\right)$ and $\left(E_{Ga2p_{3/2}}^{{Ga}_{2}O_{3}}-E_{VBM}^{Ga_{2}O_{3}}\right)$ represent the valence band maxima (VBM) relative to core levels for the doped Si substrate and Ga_2_O_3_ nanowires, respectively. Figure 5a displays Ga 2p3/2 core levels and valence bands of Ga_2_O_3_ nanowires. Linear extrapolation of the valence band edge yields a VBM position of 3.7 eV, with Ga 2p3/2 binding energy at 1118.1 eV. Correspondingly, Figure 5b shows p-type Si substrate spectra where Si 2p binding energy (99.2 eV) and VBM (0.4 eV) were determined identically. Interface-specific photoelectron spectra (Ga 2p3/2 and Si 2p) are presented in Figure 5c, yielding $∆E_{CL}$ = 1012.4 eV for sample P1. In general, the conduction band offset (CBO) can be determined by the following equation: ${∆E}_{c}=\left(E_{g}^{{Ga}_{2}O_{3}}-E_{g}^{Si}\right)-∆E_{v}$, where $E_{g}^{{Ga}_{2}O_{3}}$ and $E_{g}^{Si}$ are the band gap energies of Ga_2_O_3_ and Si, respectively. In this work, the $∆E_{v}$ of the P1 sample was calculated to be 3.0 eV, the band gap energy of Ga_2_O_3_ was 4.7 eV, and the band gap energy of Si was 1.12 eV at room temperature. Therefore, the CBO was calculated to be 0.58 eV. The Ga 2p3/2 CL and valence band spectra of the Ga_2_O_3_ nanowires are presented in Figure 5d, while Figure 5e displays the corresponding Si 2p CL and valence band for the n-type Si substrate. Analysis of the Ga 2p3/2 and Si 2p photoelectron emission spectra acquired at the Ga_2_O_3_/Si interface (Figure 5f) yielded valence band offset ($∆E_{v}$) and conduction band offset (${∆E}_{c}$) values of 3.2 eV and 0.38 eV, respectively, for the N1 sample. Using these calculated offsets, the energy band diagrams shown in Figure 5g,h were constructed.

When an n-type semiconductor interfaces with an electrolyte, upward energy band bending occurs at the Ga_2_O_3_/electrolyte junction due to electron transfer toward the electrolyte to establish electrochemical equilibrium [32]. This band bending generates a built-in electric field that enhances the separation of photogenerated carriers. At the interface, photogenerated holes react with hydroxide ions. Concurrently, electrons migrate through Ga_2_O_3_ nanowires and the substrate to the external circuit, ultimately reaching the Pt counter electrode [15,38,39]. Consistent with prior characterization, Ga_2_O_3_ nanowire (NAR) detectors fabricated on p-Si substrates in the 0.5 M Na_2_SO_4_ electrolyte exhibit superior performance compared to n-Si-based systems. This enhancement originates from fundamental differences in energy-band alignment and the built-in electric field effects between pn- and nn-heterojunctions. In the p-Si/n-Ga_2_O_3_ pn-junction (Figure 5g), Fermi level alignment establishes a strong built-in electric field (E_bi_) oriented from n-Ga_2_O_3_ to p-Si. Under UV illumination, photogenerated electron–hole pairs experience enhanced separation and transport driven by Ebi. With the conduction band offset ΔEC = 0.58 eV, the electric field efficiently drives electrons across the heterointerface into p-Si. Conversely, the large valence band offset (ΔE_V_ = 3.0 eV) impedes hole transport from p-Si to Ga_2_O_3_. This asymmetric carrier transport suppresses recombination, enabling high-efficiency carrier separation and significant photocurrent generation [40]. For the n-Si/n-Ga_2_O_3_ nn-junction (N1 sample), the weaker E_bi_ and smaller ΔE_C_ (0.38 eV) result in an insufficient electron injection driving force. Concurrently, poor photogenerated carrier separation enhances electron-hole recombination probability, yielding attenuated photocurrent signals and compromised detection performance.

**Figure 6:**
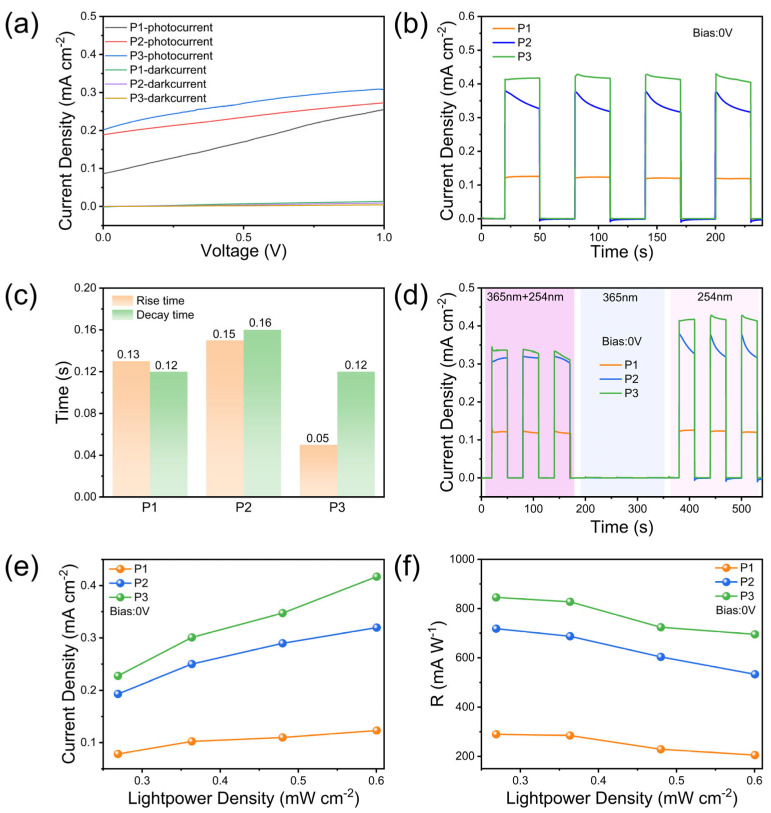
(**a**) I-V curves at 0–1 V for different carrier concentration substrate samples (P1, P2, P3). (**b**) Comparison of photocurrent densities at 0 V bias, 0.6 mW/cm^2^. (**c**) Comparison of rise/decay time. (**d**) Photocurrent densities at different optical wavelengths. (**e**) Photocurrent densities at different light intensities and (**f**) the corresponding computed photoresponsivity.

Given the significant impact of the carrier type on device performance, carrier concentration effects warrant systematic investigation. Both P2 and P3 devices exhibit exceptional photoresponsivity under 0 V bias (Figure 6a), mirroring P1’s performance. This consistency confirms the universal applicability of the self-powered operation mechanism in p-Si/n-Ga_2_O_3_ heterojunctions. Current–time characterization (Figure 6b) reveals a photocurrent density of 0.40 mA/cm^2^ for P3 under 254 nm illumination, representing a 100% enhancement over P1. This improvement originates from optimized energy-band alignment in high carrier concentration substrates, which enhances carrier transport efficiency. Transient response analysis (Figure 6c) demonstrates τᵣ = 0.05 s for P3 under 254 nm illumination, corresponding to reductions of 61.5% and 66.7% versus P1 and P2, respectively. The accelerated rise kinetics are attributed to enhanced carrier separation under strong built-in electric fields, while the shortened decay time (τd = 0.12 s) reflects the effective suppression of interface recombination centers through heterojunction hole-blocking effects. Crucially, Figure 6d confirms that P2 and P3 maintain the exceptional solar-blind UV selectivity observed in P1 during self-powered operation. Figure 6e confirms quasi-linear photocurrent density growth with irradiance for both devices under 0 V bias, consistent with P1 behavior. P3 demonstrates superior charge transport characteristics: photocurrent density scales linearly from 0.08 to 0.42 mA/cm^2^ (Δ = 425%) as irradiance increases from 0.27 to 0.6 mW/cm^2^, significantly exceeding P1 (0.08→0.12 mA/cm^2^, Δ = 50%) and P2 (0.19→0.32 mA/cm^2^, Δ = 68%). The responsivity decay kinetics in Figure 6f reveal carrier recombination dynamics. The peak R-value of 845.3 mA/W for P3 at 0.27 mW/cm^2^ corresponds to 191.8% enhancement versus P1. This improvement is attributed to p^+^-Si substrate-induced interface dipole optimization, which reduces the Schottky barrier height. Notably, the power-law decay of R with increasing light intensity aligns with the transport mechanism dominated by the space-charge-limited current (SCLC), providing quantitative theoretical support for the bandgap engineering design of high-sensitivity sun-blind ultraviolet detectors [41,42].

Table 2 benchmarks the key performance metrics of state-of-the-art solar-blind ultraviolet (SBUV) photodetectors, including the responsivity and response time. The developed β-Ga_2_O_3_/electrolyte photodetector demonstrates exceptional dual advantages: ultrahigh responsivity at 254 nm wavelength and sub-second response kinetics under zero-bias operation. These results validate the practical viability of this photovoltaic detector module for underwater solar-blind UV detection applications.

## 4. Conclusions

In summary, well-crystallized β-Ga_2_O_3_ NRAs have been synthesized on silicon substrates by a simple one-step CVD technique. The PDs prepared on p-Si substrate with a carrier concentration of 6.48 × 10^17^ cm^−3^ exhibit an excellent responsivity of 845.3 mA/W under 254 nm illumination and 0 V bias, and the rise/decay times are 0.05 s/0.12 s. The β-Ga_2_O_3_ NARs PDs exhibit multi-periodicity and good long-term stability. The excellent performance can be attributed to the additional built-in electric field at the interface of the high carrier concentration p-type silicon substrate with the β-Ga_2_O_3_ nanowires, which promotes the effective separation of photogenerated electron–hole pairs and further prevents the fast complexation of carriers. The results demonstrate the stable operation capability of underwater photodetectors based on β-Ga_2_O_3_ NARs at 0 V bias, the high sensitivity detection performance, and the wide range of applications, revealing the great potential of this device in the field of SBUV underwater detection.

## Figures and Tables

**Table 1:** The photocurrent density of the samples at 254 nm and 365 nm, and their rejection ratio.

Sample	I_254 nm_ (mA/cm^2^)	I_365 nm_ (mA/cm^2^)	R_rej_
N1	0.049	3.8 × 10^−4^	129
P1	0.130	6.3 × 10^−4^	206
P2	0.340	2.8 × 10^−4^	1214
P3	0.420	9.3 × 10^−4^	452

**Table 2:** Comparison of the performance of solar-blind UV semiconductor/electrolyte structured photodetectors.

Photodetector	Electrolyte	Condition	Bias	Photoresponsivity (mA/W)	Rise/Decay Time (ms)	Ref.
β-Ga_2_O_3_ NRAs	Na_2_SO_4_	254 nm	0 V	3.81	290/160	[15]
α-Ga_2_O_3_ NRAs	Na_2_SO_4_	254 nm	0 V	11.34	1510/180	[16]
p-AlGaN/n-GaN	NaOH	255 nm	0 V	15.5	121/151	[4]
Ga_2_O_3_-Al_2_O_3_	NaOH	254 nm	0 V	0.174	100/100	[19]
Amorphous Ga_2_O_3_/carbon fiber paper	Na_2_SO_4_	254 nm	0 V	12.90	150/130	[18]
β-Ga_2_O_3_ single crystal	NaCl	213 nm	0.8 V	25.1	76/83	[34]
β-Ga_2_O_3_ NRAs on p-Si (2.68 × 10^15^ cm^−3^)	Na_2_SO_4_	254 nm	0 V	289.7	130/120	This work
β-Ga_2_O_3_ NRAs on p-Si (6.48 ×10 ^17^ cm^−3^)	Na_2_SO_4_	254 nm	0 V	845.3	50/120	This work

## Data Availability

The data are available on reasonable request from the corresponding author.
